# Diversity in nonlinear responses to soil moisture shapes evolutionary constraints in *Brachypodium*

**DOI:** 10.1093/g3journal/jkab334

**Published:** 2021-09-27

**Authors:** J Grey Monroe, Haoran Cai, David L Des Marais

**Affiliations:** 1 Department of Plant Sciences, University of California at Davis, Davis, CA 95616, USA; 2 Department of Civil and Environmental Engineering, Massachusetts Institute of Technology, Cambridge, MA 02139, USA; 3 The Arnold Arboretum of Harvard University, Boston, MA 02130, USA

**Keywords:** *Brachypodium*, drought, evolutionary constraint, function-valued traits, nonlinearity, phenotypic plasticity, water availability

## Abstract

Water availability is perhaps the greatest environmental determinant of plant yield and fitness. However, our understanding of plant-water relations is limited because—like many studies of organism-environment interaction—it is primarily informed by experiments considering performance at two discrete levels—wet and dry—rather than as a continuously varying environmental gradient. Here, we used experimental and statistical methods based on function-valued traits to explore genetic variation in responses to a continuous soil moisture gradient in physiological and morphological traits among 10 genotypes across two species of the model grass genus *Brachypodium*. We find that most traits exhibit significant genetic variation and nonlinear responses to soil moisture variability. We also observe differences in the shape of these nonlinear responses between traits and genotypes. Emergent phenomena arise from this variation including changes in trait correlations and evolutionary constraints as a function of soil moisture. Our results point to the importance of considering diversity in nonlinear organism-environment relationships to understand plastic and evolutionary responses to changing climates.

## Introduction

Soil water availability is an important environmental factor in ecology and agriculture, acting as a major determinant of plant fitness and yield (Juenger 2013; Greenham *et al.* 2017). As such, considerable effort has been placed on studying plant responses to drought, often defined conceptually and experimentally as an environmental condition of abnormally elevated aridity resulting in decreased plant performance (Passioura 1996). Most of the research on drought responses and tolerance strategies, including in *Brachypodium*, the focal system of this work, has involved comparisons between discrete soil water levels—control and water-limited (Des Marais *et al.* 2012; Edwards *et al.* 2012; El-Soda *et al.* 2014; Vasseur *et al.* 2014; Greenham *et al.* 2017). Yet, as with most environmental factors experienced by all organisms, soil moisture is complex and multidimensional, with fluctuations varying continuously in timing, duration, and degree. Here we investigate trait responses to one important dimension of soil moisture variability—degree—with experimental and statistical approaches treating soil moisture content as a continuous variable rather than a set of fixed levels.

Quantitative genetic variation in drought resistance traits have been observed in natural populations and laboratory model systems. In particular, natural populations of Brassicaceae species (including *Arabidopsis thaliana*) and *Brachypodium* harbor variation both constitutive and plastic traits mediating plant-water relations, including water use efficiency (WUE) (Des Marais *et al.* 2012, 2017; Edwards *et al.* 2012; Greenham *et al.* 2017), leaf chemistry (Kesari *et al.* 2012; Des Marais *et al.* 2017), leaf anatomy (Skirycz *et al.* 2011; Verelst *et al.* 2013; Dittberner *et al.* 2018), root-shoot biomass partitioning (Des Marais *et al.* 2012, 2017), and many others (Verslues and Juenger 2011; Edwards *et al.* 2012; Juenger 2013; Luo *et al.* 2016; Yarkhunova *et al.* 2016; Lenk *et al.* 2019). Among the many plant traits that can and have been measured, few have been studied more extensively than specific leaf area (SLA—often reported as its inverse, leaf mass per area or LMA). SLA provides a description of leaf architecture that is central to the leaf economics spectrum, a theory which seeks to explain variation in leaf physiological strategies, from more conservative (low SLA) to more productive (high SLA) (Wright *et al.* 2004). However, for all of these traits, the shape of plastic responses to variation in water availability remain largely unmeasured.

Still less is known about how trait variation and relationships between traits change across continuous environmental gradients (but see Robinson *et al.* 2009). Genetic variation for traits can be higher, for example, in less frequently encountered environmental conditions (Gibson and Dworkin 2004; Schlichting 2008; Paaby and Rockman 2014). Describing this structure is important as trait variances and covariances influence evolutionary constraints. For example, if total genetic variance in trait space changes depending on the environment, then the capacity to respond to selection will vary accordingly, with reduced responses to selection under conditions where genetic variance is lower and vice versa. Similarly, if trait covariances depend on environment (Wood and Brodie 2015), conditions which increase trait covariances may limit evolutionary potential across a range of environments by reducing the effective axes of variation (Levins 1968; Lande and Arnold 1983; Via and Lande 1985; Kingsolver and Gomulkiewicz 2003; Gomulkiewicz *et al.* 2018). These phenomena are made more complex by the possibility that the relationship between trait variances and covariances with the environment may be nonlinear. Investigating the genetic architecture of multiple traits is therefore useful for understanding evolution in rapidly changing environments. Fortunately, this need has been met with the development of statistical methods to reduce the dimensionality of genetic variance-covariance matrices and to produce meaningful summaries describing evolutionary constraints of multiple phenotypes simultaneously (Houle 1992; Blows 2007; Kirkpatrick 2009; Kingsolver *et al.* 2015).

To study the shape of phenotypic responses to the environment, modeling traits as mathematical functions of continuous variables, or function-valued-traits, has proven powerful (Kirkpatrick and Heckman 1989; Kingsolver *et al.* 2001; Griswold *et al.* 2008; Stinchcombe *et al.* 2012; Goolsby 2015; Gomulkiewicz *et al.* 2018). Such approaches have now been used to study diverse organisms (Gibert *et al.* 1998; Pettay *et al.* 2008; Rocha and Klaczko 2012; Mason *et al.* 2020), traits (Robinson *et al.* 2009; McGuigan *et al.* 2010; Stinchcombe *et al.* 2010), and components of the environment (Brommer *et al.* 2008; McGuigan 2009; Pearse *et al.* 2020). For plant scientists, experimental and analytical approaches leveraging the concept of function-valued-traits provide a framework for gaining a deeper understanding of plant acclimation and evolutionary adaptation to the environment.

In this study, we combine these conceptual and analytical approaches to investigate how trait plasticity, trait covariance(s), and the resulting evolutionary constraints vary across soil moisture gradients. We model trait responses to a continuous gradient of soil moisture for multiple genotypes of two species of the model grass genus *Brachypodium*: annual *B. distachyon* and perennial *B. sylvaticum*. Because the shapes of trait responses cannot necessarily be known *a priori*, we use model selection among linear and nonlinear environmental predictors to estimate the response function for each trait. We then estimate genotype means at different levels of soil moisture and compute the variance-covariance parameters for all traits. Finally, we ask whether patterns of variance and covariance in drying-responsive traits in *Brachypodium* species may lead to variation in evolutionary constraint as a function of soil moisture.

## Materials and methods

### Genotypes and species

*Brachypodium* is a model genus for the genetics and genomics of C3 grasses (Brkljacic *et al.* 2011). In this study, we studied natural variation between and among two species of *Brachypodium*: the annual *B. distachyon* and the perennial *B. sylvaticum*. Both species are endemic to Eurasia, with *B. distachyon* more prevalent in seasonally dry habitats in Southern Europe, North Africa, and the Middle East, and *B. sylvaticum* showing much broader latitudinal and longitudinal range throughout Eurasia (Catalan *et al.* 2016) (Supplementary Figure S1). The different evolutionary histories, ecological environments, and life habits of these two species may have impacted patterns of genetic variation and evolvability in relation to soil moisture.

To investigate genetic variation, five genotypes of each species were studied to characterize patterns of variation in plant traits across an environmental gradient: *Brachypodium distachyon* inbred lines ABR2, Adi-10, Bd21, Bd3-1, and Koz-1 and *Brachypodium sylvaticum* inbred lines Ain-1, Ast-1, Kry-1, Osl-1, Vel-1. For each species, these genotypes represent a range of geographical origins and phenotypic diversity (Steinwand *et al.* 2013; Des Marais *et al.* 2017). For example, Kry-1 is native to Krym, Ukraine while Ain-1 is native to Ain Draham, Tunisia (Steinwand *et al.* 2013). Both species are self-compatible and each of the lines used here have been maintained as inbred lines for greater than six generations (Vogel *et al.* 2009; Steinwand *et al.* 2013); as such, experimental replicates may be considered nearly isogenic.

### Plant growth and dry down experiment

Plant growth and experimental soil dry down were performed in the greenhouses of the Arnold Arboretum of Harvard University. To synchronize germination across genotypes within each species, seeds were placed on damp filter paper in the dark at 4°C for 14 days prior to planting. To synchronize the developmental stage at the timing of the drought treatments between the two species, *B. sylvaticum* seeds were planted thirteen days before *B. distachyon* (October 7 and 20, 2015, respectively). For each genotype, 1200 seeds were planted two to a pot and were subsequently thinned to one plant, for a total of 600 experimental plants in a randomized block design. All plants germinated within four days of sowing. Individual seeds of plants were sown in Greens Grade Profile porous ceramic rooting media (Profile Products, Buffalo Grove, IL, USA) in Deepot D40H Conetainers (650 mL; Stuewe & Sons. Tangent, OR, USA) with 2% v/v solution of DynaGro Grow liquid fertilizer (DynaGro. Richmond, CA, USA) and grown at 25°C/20°C days/nights. Ambient sunlight was supplemented to 1000 μmol/m^2^/s for 12 h/day.

In order to measure the plants at a similar developmental time point, dry down treatments began 29 and 42 days after sowing (DAS) for *B. distachyon and B. sylvaticum*, respectively, to account for the slower development of *B. sylvaticum* (Lundgren et al. in prep). Because the harvesting was divided over five consecutive days (see the section below), plants were split into five equal harvest cohorts, with each cohort containing equal numbers of each watering treatment to avoid confounding harvest day with soil moisture content. Thus, though each consecutive cohort differed in age by a single day, each experienced the dry down treatment for the same amount of time. Nevertheless, we expected the difference in age between harvest cohorts to potentially impact trait expression and we therefore included harvest day (cohort) as a covariate in subsequent models. To generate a continuous gradient of final soil moisture by the end of the dry down period, plants were split into five watering treatments, receiving 0, 4, 8, 12, 16, or 20 ml of water per day for 14 days (Supplementary Figure S2). Prior to initiating the experiment pots were weighed with dry soil ($\mathrm{mas}s_{\mathrm{dry}}$) and at the field capacity of the soil for water ($\mathrm{mas}s_{\max}$). Field capacity was determined by saturating the soil with water and then letting the water drain for 24 h; the amount of the water at this time point constitutes field capacity. During the course of the dry down, soil moisture content was calculated during the morning of day d for each pot as $\mathrm{mas}s_{d}/(\mathrm{mas}s_{\max}-\mathrm{mas}s_{\mathrm{dry}})$. The final soil moisture reflects the combined effects of water input and output through evaporation from the soil and evapotranspiration from the plants.

### Plant harvesting and phenotyping

To characterize phenotypic responses to our experimental soil moisture gradient, we measured a suite of developmental and physiological traits. Plants were harvested in five cohorts over five days at the end of the dry down period. Each day, half of the sampled plants were harvested for above and below-ground biomass, total above-ground green area, $\delta C_{13}$, N content, C content. The other half were harvested and assessed for SLA and relative water content (RWC). Above-ground leaf area was estimated by laying freshly harvested plants flat between plates of clear acrylic and imaging with a Nikon 5300 digital camera at a fixed distance with a 35 mm Nikkor lens. Total green pixels were counted for each image with Easy Leaf Area (Easlon and Bloom 2014) (https://github.com/heaslon/Easy-Leaf-Area) with settings shown in Supplementary Figure S3. Above-ground biomass was measured after drying leaf material overnight at 60°C and then for several weeks at room temperature. Below ground biomass was measured after washing the soil matrix from roots and drying them overnight at 60°C and then for several weeks at room temperature. Above and below-ground biomass was measured after leaves and roots were dried. Leaf tissues for δ^13^C, δ^15^N, nitrogen (hereafter “N”) content, and carbon (hereafter “C”) content were ground to a fine powder and processed by the UC Davis Stable Isotope Facility. δ^13^C is a widely used proxy for WUE in plants employing C3 photosynthesis (Farquhar and Richards 1984). Here, lower (more negative) values of δ^13^C reflect a greater discrimination by RuBisCO for ^12^C and is interpreted as lower WUE over the development of the studied tissue. In controlled-environment settings such as that employed here, δ^15^N ratios largely reflect frationation of ^15^N and ^14^N during N assimilation in the plant (Craine *et al.* 2015). In this context, possible causes of variation in δ^15^N include genetic variation for enzymatic preference for the two N isotopes, or slight variation in the relative uptake of nitrate or ammonia from the synthetic fertilizer (which may have different isotopic ratios by chance) by the plants along the soil moisture gradient. While we have no specific hypothesis about δ^15^N we include it here as an additional trait that may show patterns of response. SLA was calculated by scanning the two youngest fully emerged leaves with a 1 cm^2^ red square. Leaf area in mm^2^ was calculated from these same images using Easy Leaf Area. SLA was calculated as $\mathrm{leaf}\;\mathrm{area}/\mathrm{biomas}s_{\mathrm{dry}}$. These leaves were also used to calculate RWC. Prior to drying, fresh leaves were weighed ($\mathrm{biomas}s_{\mathrm{fresh}}$) and then submerged under water in 15 mL falcon tubes for several hours. They were then weighed ($\mathrm{biomas}s_{\mathrm{turgid}})$, oven-dried overnight, and weighed again ($\mathrm{biomas}s_{\mathrm{dry}}$). RWC was calculated as $\left(\mathrm{biomas}s_{\mathrm{fresh}}-\mathrm{biomas}s_{\mathrm{dry}})/(\mathrm{biomas}s_{\mathrm{turgid}}-\mathrm{biomas}s_{\mathrm{dry}}\right)$.

## Analyses

We used R for all statistical analyses. Code and data to generate this manuscript can be found at https://github.com/greymonroe/brachypodium_fvt and Zenodo (10.5281/zenodo.4446263).

### Function-valued traits

For the purposes of modeling phenotypic responses to variation in soil moisture content, we considered soil moisture content as the final soil moisture on day 14 of the dry down period for each plant, referred to in figures as $\mathrm{Soil}\;\mathrm{moisture}({\%}_{\mathrm{final}})$. A major challenge in studying function-valued traits is model selection. That is, identifying the functions that best describe the curvature (or lack thereof) in the shape of phenotypic responses to environmental gradients. Quadratic and natural splines have been suggested as potential functions to model nonlinearities (Meyer 2005), but assuming the appropriate function is problematic. Akaike information criterion (AIC) selection based on contrasting multiple complex models offers an effective means to balance predictability with over-fitting (Griswold *et al.* 2008; Bolstad *et al.* 2010; Gomulkiewicz *et al.* 2018). Because we sampled only five genotypes per species and because the shape of reaction norms for each trait in relation to soil moisture was not known *a priori*, part of our analyses was aimed exploring models with different nonlinear predictors with AIC from a full model which necessitated treating genotype as a fixed effect (Van Eeuwijk 1995). We therefore treat genotype as a fixed effect in our model, which differs from random regression used elsewhere where genotypes are treated as random effects (Nussey *et al.* 2007). Thus, we began with the full fixed-effects linear regression model for traits as described below.

$$\mathrm{Trait}=H+G+E+E^{2}+s(E{)}_{df=2}+G*E+G*E^{2}+G*s(E{)}_{df=2}$$

where $H=\mathrm{harvest}\;\mathrm{day},\;G=\mathrm{genotype},\;E=\mathrm{soil}\;\mathrm{moisture},\;E^{2}=\mathrm{quadratic}\;\mathrm{parameter},\;s(E{)}_{df=2}=\mathrm{second}\;\mathrm{degree}\;\mathrm{natural}\;\mathrm{splin}e\;\mathrm{parameter}$

We then selected a model for each trait using stepwise AIC model selection with the stepAIC function from the package MASS (Venables and Ripley 2002) in R with the “direction” parameter set to “both.” The two species were analyzed separately to avoid biases introduced by enforcing the same model on species with different sizes, developmental trajectories, and evolutionary histories.

### Genetic correlations as a function of soil moisture content

We calculated trait correlations at different levels of soil moisture to characterize how genetic correlations between traits vary as a function of soil moisture content. Predicted genotypic means for each trait were calculated at 20 levels of soil moisture content (from 30% to 100% gravimetric water content) based on the model chosen by AIC (see above). Pearson correlation coefficients between genotype means were calculated at each level of soil moisture within each species.

### Plasticity through multidimensional trait space

We quantified total plasticity through multidimensional trait space as a function of soil moisture by first scaling each trait to a mean of 0 and standard deviation of 1. We then calculated the Euclidean distance matrix between genotype means at all soil moisture levels. We then estimated total plasticity, measured as Euclidean distance between trait values, between consecutive soil moisture levels for each genotype. m is a vector of length 20 evenly spaced soil moisture measurements from 30% to 100%:

$$m=(m_{1},\ldots ,m_{20})=(30\%,\ldots ,100\%)$$

If $p_{g,i}=(p_{g,1},\ldots ,p_{g,n})$ and $p_{g,i+1}=(p_{g,i+1,1},\ldots ,p_{g,i+1,n})$ are two consecutive (in terms of soil moisture) points in n dimensional trait space of scaled genotype means, total plasticity (Euclidean distance) for genotype g across n traits at soil moisture level i is calculated as

$$\Delta T_{g,i}=\sqrt{\sum_{j=1}^{n}{\left(p_{g,i,j}-p_{g,i+1,j}\right)}^{2}}$$

Finally, to ask if total plasticity differed between the two species, at each level of soil moisture, we then compared the mean ΔT between *B. sylvaticum* and* B. distachyon* by *t*-tests. To visualize plasticity of each genotype through multivariate trait space further, we performed a principal component analysis from the matrix of scaled genotype trait means using the *prcomp* function in R.

### Analysis of evolutionary constraints among traits

We calculated several statistics summarizing evolutionary constraints as described in Kirkpatrick (2009). First, for each species, we approximated the G matrix of genetic covariances by calculating variances and covariances between mean scaled genotype trait means (best linear unbiased estimates) at different levels of soil moisture. We then calculated, using the *prcomp* function in R, the eigenvalues of each mean standardized (trait values divided by mean) G matrix, $\lambda_{i}$. From these, we then estimated the *number of effective dimensions*, $n_{D}$, equal to the sum of the eigenvalues divided by the largest eigenvalue. This value can range from 1 (all genetic variation in a single dimension; high constraint) to the total number of traits in the G matrix (no genetic covariances; low constraint) (Levins 1968; Lande and Arnold 1983; Via and Lande 1985; Kingsolver and Gomulkiewicz 2003; Gomulkiewicz *et al.* 2018).

$$n_{D}=\sum_{i=1}^{n}\lambda_{i}/\lambda_{l}$$

We also estimated the *maximum evolvability*, $e_{\max}$, equal to the square root of the largest eigenvalue, $\lambda_{l}$ (Houle 1992; Kirkpatrick 2009). This is the mutlivariate equivalent of Houle’s (1992) measure of evolvability of a single trait.

$$e_{\max}=\sqrt{\lambda_{l}}$$

Finally, we estimated the *total genetic variance* (Kirkpatrick 2009), equal to the sum of the eigenvalues of G. This value reflects the capacity to respond to selection across multiple traits.

$$v_{T}=\sum_{i=1}^{n}\lambda_{i}$$

By calculating these estimates of constraint in each species at different levels of soil moisture we could observe how constraints varied across soil moisture gradients and between species.

## Results

### The dry down experiment resulted in a continuous soil moisture gradient

**Figure 1:**
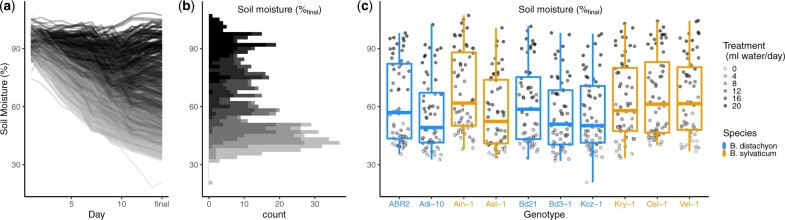
Effect of the experimental dry down on soil water content. (A) Time series of gravimetric soil moisture for all pots during the 14-day dry down period. (B) Distribution of final (day 14) soil moisture content across all pots. The data are distinguished by color according to the watering treatment. (C) Final soil moisture content by genotype.

Across the six watering treatments, combined with stochastic variation in water capacity of pots (Supplementary Figure S4), the dry down period resulted in a continuous environmental gradient of final soil moisture, albeit with a higher frequency of plants near the driest extreme of soil moisture variation (Figure 1). This gradient provides the basis for analyzing phenotypes in relation to soil moisture treated as a continuous gradient rather than a limited set of discrete factors.

**Figure 2:**
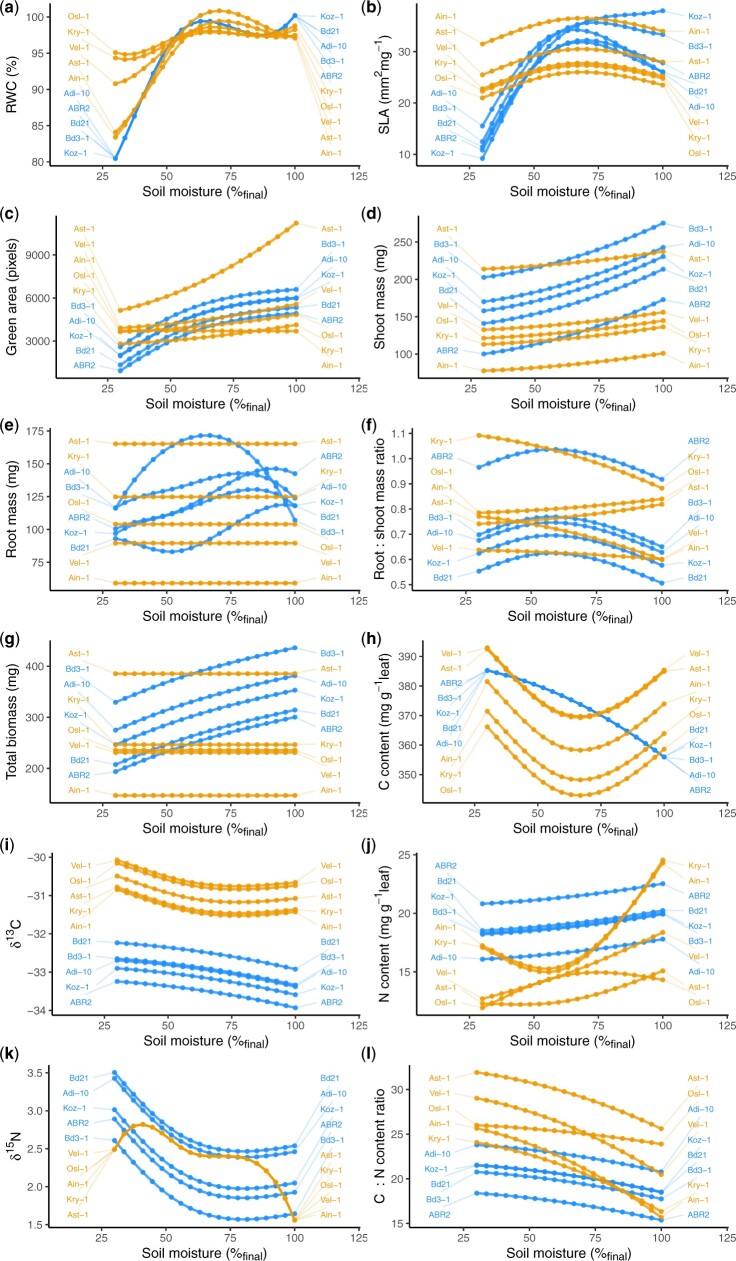
Variation in phenotypic responses to soil moisture gradient as function-valued traits. Points and the lines connecting them indicate predicted values at different levels of gravimetric soil moisture content from 30% to 100%. *B. sylvaticum* genotypes are colored orange and *B. distachyon* blue. (A) RWC, (B) SLA, (C) Green Area, (D) Shoot Mass, (E) Root Mass, (F) Root: Shoot, (G) Biomass, (H) C content, (I) ^1^^3^C, (J) N content, (K) ^1^^5^N, and (L) C:N ratio.

The observed reduction in leaf RWC under the driest conditions in both *B. distachyon* and* B. sylvaticum* indicates that at this extreme, plants were physiologically stressed (Figure 2A). Mean leaf RWC for plants in the 10% tail of soil water content was 85.21% which is drier than that observed in the less severe soil drying conditions in an earlier study (Des Marais *et al.* 2017). Additional visual observations made during the experiment such as leaf rolling, another symptom of dehydration stress, were evident in plants at the lowest water treatment by the end of the dry down period.

### Nonlinearity in trait responses to soil moisture is common

We evaluated the degree to which traits showed linear or nonlinear responses to soil moisture using an AIC model selection approach from a full model which included quadratic and natural spline parameters relating soil moisture content to plant phenotypes. We observed nonlinear components (quadratic, spline, or both) in the final models for all of the traits which included an environmental (water content) predictor (Table 1, Figure 2, Supplementary Tables S1 and S2). In *B. distachyon*, all of the traits included at least one nonlinear environmental predictor. In contrast, total biomass and root mass did not include an environmental predictor in selected models in *B. sylvaticum*.

**Table 1:** Model selection “x” indicates parameters chosen in best minimum AIC model. H = harvest day, G = genotype, E = final soil moisture, E^2^ = quadratic parameter, s(E) = 2nd degree natural spline parameter

	G	E	E^2^	s(E)	H	G*E	G*E^2^	G*s(E)
RWC—*B. distachyon*	—	—	x	x	x	—	—	—
RWC—*B. sylvaticum*	x	—	x	x		—	—	x
SLA—*B. distachyon*	x	—	x	x	x	—	x	—
SLA—*B. sylvaticum*	x	—		x		—	—	—
Green Area—*B. distachyon*	x	—		x	x	—	—	—
Green Area—*B. sylvaticum*	x	—	x	—	x	—	x	—
Shoot Mass—*B. distachyon*	x	—	x	—	x	—	—	—
Shoot Mass—*B. sylvaticum*	x	—	x	—	x	—	—	—
Root Mass—*B. distachyon*	x	—	x	x	x	—	—	x
Root Mass—*B. sylvaticum*	x	—	—	—	—	—	—	—
Root: Shoot—*B. distachyon*	x	—	—	x	x	—	—	—
Root: Shoot—*B. sylvaticum*	x	—	x	—	x	—	x	—
Biomass—*B. distachyon*	x	—	—	x	x	—	—	—
Biomass—*B. sylvaticum*	x	—	—	—	x	—	—	—
C content—*B. distachyon*	—	—	x	—	—	—	—	—
C content—*B. sylvaticum*	x	—	—	x	—	—	—	—
d13c—*B. distachyon*	x	—	x	—	x	—	—	—
d13c—*B. sylvaticum*	x	—	—	x	x	—	—	—
N content—*B. distachyon*	x	—	x	—	x	—	—	—
N content—*B. sylvaticum*	x	—	—	x	—	—	—	x
d15n—*B. distachyon*	x	—	—	x	x	—	—	—
d15n—*B. sylvaticum*	—	—	x	x	x	—	—	—
C: N ratio—*B. distachyon*	x	—	x	—	x	—	—	—
C: N ratio—*B. sylvaticum*	x	—	x	—		—	x	—

**Figure 3:**
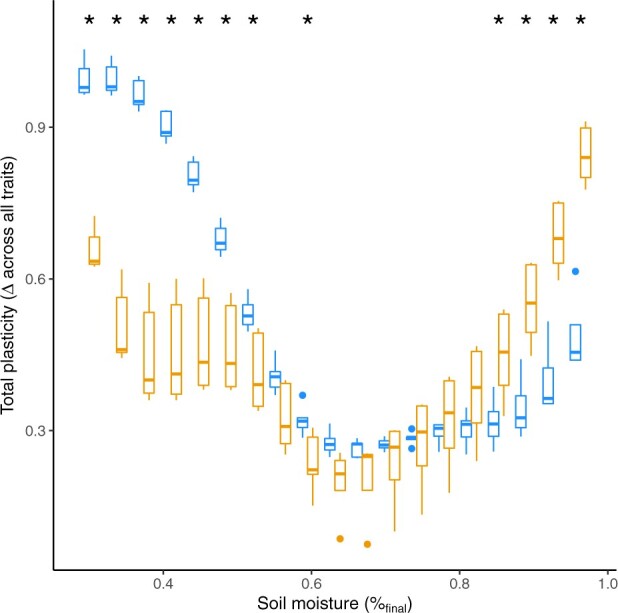
Plasticity through multivariate trait space. Plasticity across all traits was calculated as distance between scaled phenotype for each genotype between different levels of soil moisture. Box plots indicate species median and 25th and 75th percentiles with whiskers extending to 1.5 times the interquartile range. “*” indicates significant differences between species (*t*-test, α<0.05). *B. sylvaticum* is colored in orange and *B. distachyon* blue.

When considering plasticity across multidimensional trait space, it appears that most of the variation in *B. distachyon* is attributable to responses to low soil moisture (Figure 3, Supplementary Figure S5). In contrast, *B. sylvaticum* was more responsive to extreme wet conditions than *B. distachyon*. We observed, particularly in *B. sylvaticum*, that phenotypes were similar between extreme dry and extreme wet soil moisture contents (Supplementary Figure S5). This similarity may be explained by the quadratic parameters of trait functions where the curvature of trait responses can lead to similar phenotypes at both environmental extremes.

### Most traits show significant genetic variation

We also tested whether there was significant natural variation for the traits measured between genotypes in each species by looking at the parameters in the final model for each trait. Interestingly, across species, most traits (21/24) included a genotype term in the final model, indicating significant differences between genotypes in magnitude of traits across all levels of soil moisture (Table 1, Figure 2). Though not formally tested, for other traits there are clear distinctions between the two species. For example, $\delta C_{13}$ was considerably higher in *B. sylvaticum* (Figure 2). For SLA, while *B. distachyon* showed a strong response to soil moisture, especially under the driest conditions, SLA in *B. sylvaticum* was much less responsive to soil moisture. In contrast, *B. sylvaticum* appears to show a more dramatic response in leaf composition estimated by N content and C: N ratio. We note, too, that most of the traits included an effect of harvest cohort; this is expected due to differences in age (up to 5 days, 9%–11% total) of different harvest cohorts.

### Several traits show interactions between genotype and nonlinear responses to soil moisture

Significant interactions between genotype and environmental parameters in a final model indicate the presence of genetic variation for plasticity (GxE) (Via and Lande 1985; Des Marais *et al.* 2013). For those GxE interactions where the environmental parameter is nonlinear, significant GxE indicates genetic variation for the shape of reaction norms. SLA and root mass showed a significant interaction between genotype and soil moisture in *B. distachyon*. In *B. sylvaticum*, RWC, N content, leaf green area, C: N ratio, and root to shoot ratio all showed interactions between genotype and soil moisture (Table 1, Figure 2). In each of these cases, the interaction between genotype and environment involved a nonlinear environmental predictor, indicating not only variation for the magnitude of plasticity (*i.e.*, slope) but also variation in the shape of responses.

### Correlations between traits change as a function of soil moisture, often in a nonlinear fashion

**Figure 4:**
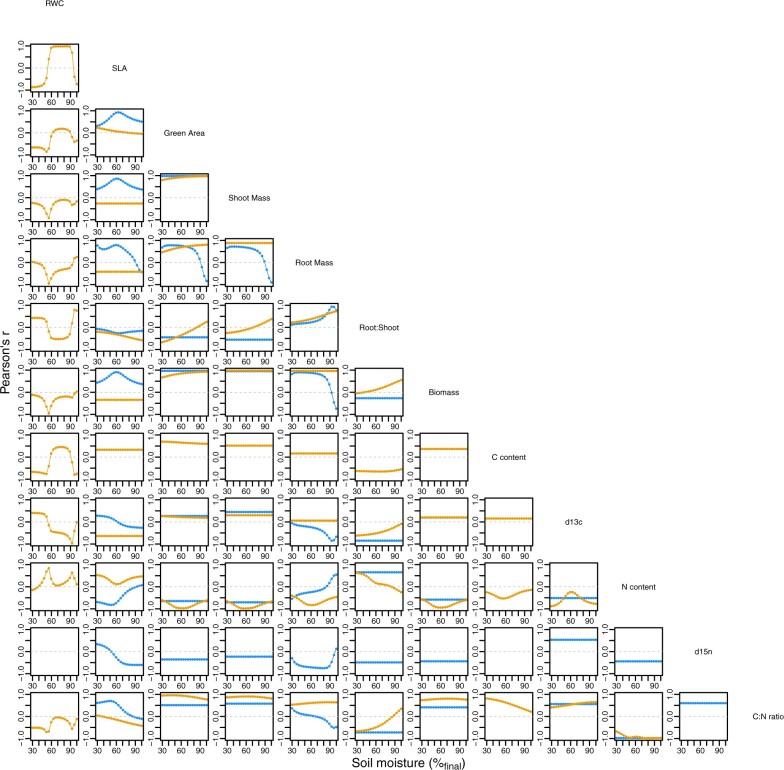
Relationships between trait correlations and soil moisture content. Each point is a correlation coefficient calculated across five genotypes between the intersection of the two traits labeled on the diagonal. Correlations were calculated among genotypes by species (blue = *B. distachyon*, orange = *B. sylvaticum*). Correlations are not shown for traits in species where genotype was not included in final model (Table 1).

We calculated correlations between genotype trait means across soil moisture for traits where genotypic differences were observed (*i.e.*, genotype predictor in trait models). Certain traits were tightly correlated regardless of environment. For example, correlations near 1 were observed between biomass and green area in both species across soil moisture. More complex relationships between trait correlations and soil moisture were observed in other trait combinations. For traits with genotype by nonlinear environment interactions (Table 1), trait correlations showed nonlinear relationships with soil moisture as well (Figure 4). Because more of these interactions were found in *B. sylvaticum*, the number of trait combinations showing nonlinear relations between correlations and soil moisture appears to be higher than in *B. distachyon* (Figure 4). The correlation between C: N ratio and root: shoot ratio in *B. sylvaticum*, for example, varied from approximately 0.3 under the wettest environment to approximately −0.7 under the driest environment.

### Evolutionary constraints differ as a function of soil moisture and show contrasting patterns between *Brachypodium* species

**Figure 5:**
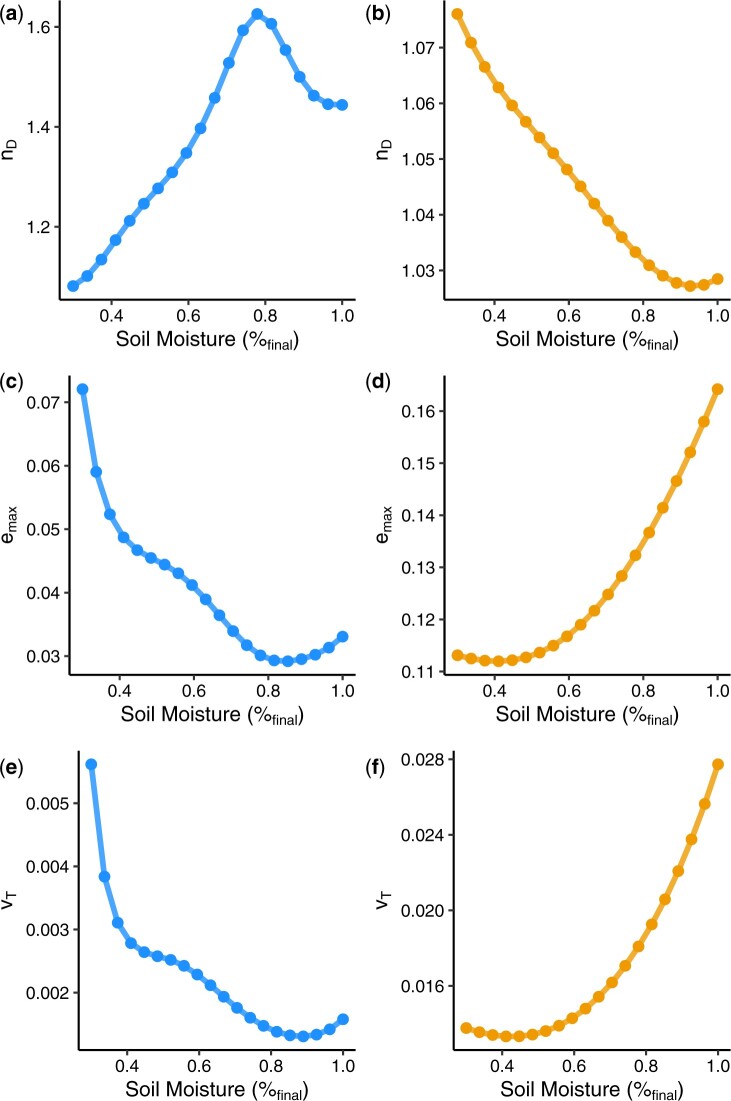
Contrasting patterns of evolutionary constraint between *B. distachyon* and* B. sylvaticum*. Summary statistics of evolutionary constraint as a function of soil moisture in *B. distachyon* (blue) and *B. sylvaticum* (orange). (A,B) The number of effective dimensions, $n_{D}$, estimates the number of unconstrained axes of variation. (C,D) The maximum evolvability, $e_{\max}$, corresponds to the square root of the largest eigenvalue of the genetic covariance matrix. (E,F) The total genetic variance, $v_{T}$, is equal to the sum of the eigenvalues of the genetic covariance matrix.

To assess evidence of evolutionary constraint on the sampled traits, we approximated and analyzed parameters of the genetic covariance matrix, G, in each species across the soil moisture gradient. These analyses revealed contrasting patterns of evolutionary constraint both in relation to soil moisture and between *B. distachyon and B. sylvaticum* (Figure 5). In *B. distachyon*, the number of effective dimensions ($n_{D}$, which estimates the number of axes of variation unconstrained by covariance) was lower when soils were drier. In contrast, $n_{D}$ was lower in *B. sylvaticum* when soils were wetter. The maximum evolvability ($e_{\max}$, variance through largest eigenvector of multidimensional trait space) also showed opposite trends between the two species. Whereas in *B. distachyon* $e_{\max}$ was highest under the driest conditions, in *B. sylvaticum* $e_{\max}$ was highest under the wettest conditions. The same trend was seen in total genetic variance ($V_{T}$, which summarizes all genetic variance through multidimensional trait space).

The similarity between the $e_{\max}$ and $V_{T}$ reflects the underlying covariance between traits with the first eigenvector explaining a large proportion of the covariance (>90%). Thus, changes in total covariance are largely driven by changes in variance explained across the first eigenvector. We manually checked the eigenvector biplots and did not find a single trait that was disproportionately influencing the measures of constraint. This is consistent with the $n_{D}$ being close to 1 for both species across the entire soil moisture gradient, indicating a single large dimension of covariance among traits.

## Discussion

### Nonlinearity in soil moisture response is common in *Brachypodium*

We found significant nonlinearity in response to a soil moisture gradient for all measured traits in at least one of the two species sampled. The best-fit function for some traits was quadratic, while other traits showed more complex responses to the environment which were best fit by a spline function. These results offer new insights with respect to the study of organismal response to the environment. By focusing on the curvature of phenotypic response as the explicitly modeled trait, we avoid contrasts of trait values expressed at arbitrary levels of soil water content which may obscure different thresholds of response among the diverse genotypes under study. SLA in *B. distachyon* exhibits this pattern, as two accessions show a threshold-like response in decreasing SLA as soils become drier, and three accessions express their highest SLA at intermediate soil water content. Leaf N content (on a leaf-dry mass basis) in *B. sylvaticum* likewise shows considerable diversity of response with two accessions expressing their lowest values at intermediate soil water content, one accession expressing its highest leaf N at intermediate soil water content, and one accession showing a nearly linear response along the soil water content gradient.

Nonlinearities in trait responses to soil moisture reinforce the need to consider the consequences of extreme weather events when predicting plastic responses, especially when scaling up to investigating the ecosystem effects of individual organismal responses to environmental stress (Felton and Smith 2017). As others have previously noted, if organism responses to the environment show nonlinear curves, then changes in the environment may result in greater or lesser responses than expected based on linear response curves alone (Nussey *et al.* 2007; Brommer *et al.* 2008; Visser 2008; Gienapp and Brommer 2014). This suggests, for example, that responses of traits such as SLA to increasing aridity and drought may depend on the severity of environmental change in a threshold-like fashion. Moreover, because traits like SLA have impacts on broader processes including carbon cycling, carbon models incorporating plastic and evolutionary responses may benefit from information about the (non)-linearity of trait responses to environmental change (Donovan *et al.* 2014; Monroe *et al.* 2018; Walker *et al.* 2019).

### Implications for evolution of *Brachypodium*

Leaf N and SLA are two axes of the classic Leaf Economic Spectrum (Wright *et al.* 2004) and so the contrasting responses of these traits between the annual *B. distachyon* and perennial *B. sylvaticum* may reflect broader differences in their life history strategies. We recently reviewed evidence for physiological, anatomical, and developmental differences between herbaceous annual and perennial species, finding support for generally higher SLA in annuals, befitting a generally resource-acquisitive strategy (Lundgren and Des Marais 2020). Garnier (1992) argued that small changes in leaf anatomy (*e.g.*, SLA) will likely have large effects on plant growth rate and resource use and could therefore tip the balance between perennial and annual strategies.

We also found that signatures of evolutionary constraint differ along our imposed soil water content gradient, although our experimental design precludes formal contrasts between the two species. Specifically, we find evidence of the highest evolvability in multitrait space (measured by $e_{\max}$ and $v_{T}$) in *B. distachyon* under the driest soils. In contrast, *B. sylvaticum* exhibited evidence of greater evolvability by the same measures under the highest soil water content studied here. However, *B. distachyon* exhibits evidence of higher constraint caused by genetic covariances (low $n_{D}$ under dry conditions). These results indicate that *B. distachyon* has increased genetic variance under dry conditions as compared to wet conditions, but that natural selection may be more constrained to act on this variation due to covariance between traits. In contrast, our results suggest that *B. sylvaticum* has decreased genetic variance under dry conditions on which selection might act but that this variation is less constrained by covariance between traits.

Consistent with predictions of elevated genetic variation revealed in less frequently encountered environments (Gibson and Dworkin 2004; Schlichting 2008; Paaby and Rockman 2014), we speculate that the pattern observed could reflect the different life-history strategies of these two species. Annuality is considered a drought adaptive strategy characterized by escaping drought through phenology, by flowering before and remaining dormant as seeds during the most drought-prone seasons (Friedman and Rubin 2015; Monroe *et al.* 2019; Friedman 2020). Thus, because of their life history, populations of annuals may actually experience fewer episodes of strong selection from extreme drought, which could explain why we find elevated genetic variance under these environments. In contrast, perennials such as *B. sylvaticum* are subjected to all seasons and might therefore experience more frequent episodes of selection caused by dehydration stress, despite paradoxically being found in environments where droughts are less frequent on an annual basis. This pattern is consistent with the predictions of cryptic genetic variation revealed under environments where selection is less frequent or severe (Gibson and Dworkin 2004; Schlichting 2008; Paaby and Rockman 2014). We remain cautious about drawing this conclusion from our data as they represent a comparison between only two species and include a small sample genotypic diversity in each. However, that the difference in patterns of constraint are explained by alternative life history strategies emerges as an interesting hypothesis from these observations, one that might be addressed by future work extending the approaches here to a broader phylogenetic comparison between annual and perennial species.

### Potential to scale up investigations of function-valued responses to soil moisture

We found that genetic correlations between traits can vary over relatively small changes in soil moisture (Figure 4). Similarly, we found that signatures of evolutionary constraint varied across the environmental gradient, suggesting that responses to selection may be improved or restricted in accordance with patterns of constraint in relation to environment. Interestingly, we also found that some species may be more responsive to selection in a given environment based on patterns of constraint. However, in this study, we focus on only five genotypes per species. This reflects the inherent challenge of investigating large numbers of genotypes and high-resolution environment gradients. But larger-scale investigations are needed if results are to be extended to the large populations typical of genetic mapping studies and breeding pools. Indeed, responses to selection for drought tolerance may depend on drought severity because of these patterns in genetic correlations. And in the context of breeding, larger studies applying these approaches might be valuable for identifying conditions for which genetic correlations are aligned with breeding objectives (Bernardo 2020).

From a practical perspective, this work highlights the potential of function-valued trait approaches that may be scaled up to studying plant-water relations in more realistic field settings. In this experiment, we investigated variation in plant responses to a gradient of soil moisture using six watering levels which, in combination with random variation in water capacity of experimental pots, produced a continuous gradient of soil moisture ranging from field capacity of the soil to strongly water-limited. An important caveat in interpreting our work is that we used a homogenous media—rather than soil from *B. distachyon’s* native range—for our experiments. While our media likely offers more realistic osmotic conditions for roots than do, *e.g.*, agar plates or rapid benchtop desiccation (Kilian *et al.* 2007), the complexity of field soil would likely elicit more nuanced responses from the plant during drying (Poorter et al. 2016). Our experimental design allowed us to isolate a single variable—soil water content—without concern for confounding effects of temperature or spatial variability in the biotic or abiotic environment. In the field, variable rainfall regimes in combination with random variability between plots likely produce similar gradients of soil moisture. New sensing technologies may be useful for quantifying soil moisture or other continuous environmental gradients in an analogous fashion to that used here (Verbraeken et al. 2021), to define soil moisture quantitatively and then apply function-valued statistical approaches to contrast trait expression among genotypes. Finally, while in this experiment we used destructive phenotyping methods to measure traits, nondestructive (and high throughput) phenotyping enables measurements of yield or fitness data and thereby facilitates assessment of explicit connections between trait variation and adaptation to different degrees of soil moisture in larger experiments. Likely sample sizes much larger than those employed herein would be required in the field environment to overcome the higher phenotypic variance arising from uncontrolled environmental variance in such experiments. Nonetheless, together field phenomic approaches provide opportunities to scale up the analytical framework used here to study genetic variation for function-valued-traits in populations of diverse genotypes under realistic conditions.

## Data availability

Code and data to generate this manuscript can be found at https://github.com/greymonroe/brachypodium_fvt and Zenodo (10.5281/zenodo.4446263).

Supplementary material is available at *G3* online.

## Supplementary Material

jkab334_Supplementary_Data
