# Supplementary material for: Diversity in nonlinear responses to soil moisture shapes evolutionary constraints in Brachypodium
Source: G3 (Bethesda). 2021 Sep 27;11(12):jkab334. doi: 10.1093/g3journal/jkab334 (PMC8664479; doi:10.1093/g3journal/jkab334)
Supplement: jkab334_Supplementary_Data [file jkab334_supplementary_data.docx]

# Supplemental Information for Monroe, Cai, & Des Marais “Diversity in non-linear responses to soil moisture shapes evolutionary constraints in *Brachypodium*

Table S1:

*Final models*

|  | Df | Sum.Sq | Mean.Sq | F.value | Pr..F. | trait |
| --- | --- | --- | --- | --- | --- | --- |
| I(Day_14{}2) | 1.00000 | 1,717.38557 | 1,717.38557 | 117.86908 | 0.00000 | Relative_WC b_dist |
| ns(Day_14,df=2) | 2.00000 | 1,763.63464 | 881.81732 | 60.52164 | 0.00000 | Relative_WC b_dist |
| Harv | 4.00000 | 137.08789 | 34.27197 | 2.35218 | 0.05709 | Relative_WC b_dist |
| Residuals | 137.00000 | 1,996.12844 | 14.57028 | NA | NA | Relative_WC b_dist |
| Geno | 4.00000 | 203.20448 | 50.80112 | 4.03279 | 0.00424 | Relative_WC b_sylv |
| I(Day_14{}2)1 | 1.00000 | 378.49590 | 378.49590 | 30.04650 | 0.00000 | Relative_WC b_sylv |
| ns(Day_14,df=2)1 | 2.00000 | 431.58215 | 215.79108 | 17.13035 | 0.00000 | Relative_WC b_sylv |
| Genons(Day_14,df=2) | 8.00000 | 231.27621 | 28.90953 | 2.29495 | 0.02552 | Relative_WC b_sylv |
| Residuals1 | 116.00000 | 1,461.25261 | 12.59701 | NA | NA | Relative_WC b_sylv |
| Geno1 | 4.00000 | 140.92870 | 35.23217 | 1.75884 | 0.14122 | SLA b_dist |
| I(Day_14{}2)2 | 1.00000 | 2,414.64758 | 2,414.64758 | 120.54272 | 0.00000 | SLA b_dist |
| ns(Day_14,df=2)2 | 2.00000 | 2,327.88690 | 1,163.94345 | 58.10575 | 0.00000 | SLA b_dist |
| Harv1 | 4.00000 | 651.79297 | 162.94824 | 8.13461 | 0.00001 | SLA b_dist |
| GenoI(Day_14{}2) | 4.00000 | 337.42496 | 84.35624 | 4.21119 | 0.00310 | SLA b_dist |
| Residuals2 | 127.00000 | 2,543.99629 | 20.03147 | NA | NA | SLA b_dist |
| Geno2 | 4.00000 | 1,572.00031 | 393.00008 | 12.05355 | 0.00000 | SLA b_sylv |
| ns(Day_14,df=2)3 | 2.00000 | 162.19048 | 81.09524 | 2.48724 | 0.08735 | SLA b_sylv |
| Residuals3 | 122.00000 | 3,977.75094 | 32.60452 | NA | NA | SLA b_sylv |
| Geno3 | 4.00000 | 32,376,338.95160 | 8,094,084.73790 | 5.44806 | 0.00043 | aboveground_greenarea b_dist |
| ns(Day_14,df=2)4 | 2.00000 | 192,517,942.01652 | 96,258,971.00826 | 64.79115 | 0.00000 | aboveground_greenarea b_dist |
| Harv2 | 4.00000 | 59,381,207.50609 | 14,845,301.87652 | 9.99225 | 0.00000 | aboveground_greenarea b_dist |
| Residuals4 | 134.00000 | 199,081,240.28441 | 1,485,680.89764 | NA | NA | aboveground_greenarea b_dist |
| Geno4 | 4.00000 | 219,846,397.86896 | 54,961,599.46724 | 15.60576 | 0.00000 | aboveground_greenarea b_sylv |
| I(Day_14{}2)3 | 1.00000 | 24,060,407.68683 | 24,060,407.68683 | 6.83170 | 0.01005 | aboveground_greenarea b_sylv |
| Harv3 | 4.00000 | 77,050,113.87350 | 19,262,528.46838 | 5.46939 | 0.00043 | aboveground_greenarea b_sylv |
| GenoI(Day_14{}2)1 | 4.00000 | 33,700,623.60514 | 8,425,155.90129 | 2.39223 | 0.05412 | aboveground_greenarea b_sylv |
| Residuals5 | 126.00000 | 443,756,646.65128 | 3,521,878.14803 | NA | NA | aboveground_greenarea b_sylv |
| Geno5 | 4.00000 | 151,815.09343 | 37,953.77336 | 17.16469 | 0.00000 | Shoot_Mass b_dist |
| I(Day_14{}2)4 | 1.00000 | 66,562.14458 | 66,562.14458 | 30.10291 | 0.00000 | Shoot_Mass b_dist |
| Harv4 | 4.00000 | 102,805.02689 | 25,701.25672 | 11.62346 | 0.00000 | Shoot_Mass b_dist |
| Residuals6 | 136.00000 | 300,716.87291 | 2,211.15348 | NA | NA | Shoot_Mass b_dist |
| Geno6 | 4.00000 | 282,513.50822 | 70,628.37705 | 24.41054 | 0.00000 | Shoot_Mass b_sylv |
| I(Day_14{}2)5 | 1.00000 | 6,122.54739 | 6,122.54739 | 2.11607 | 0.14821 | Shoot_Mass b_sylv |
| Harv5 | 4.00000 | 50,339.74373 | 12,584.93593 | 4.34960 | 0.00248 | Shoot_Mass b_sylv |
| Residuals7 | 128.00000 | 370,349.46472 | 2,893.35519 | NA | NA | Shoot_Mass b_sylv |
| Geno7 | 4.00000 | 52,681.25005 | 13,170.31251 | 23.75364 | 0.00000 | Root_Mass b_dist |
| I(Day_14{}2)6 | 1.00000 | 13,857.56690 | 13,857.56690 | 24.99316 | 0.00000 | Root_Mass b_dist |
| ns(Day_14,df=2)5 | 2.00000 | 2,853.05835 | 1,426.52918 | 2.57285 | 0.08032 | Root_Mass b_dist |
| Harv6 | 4.00000 | 6,739.85641 | 1,684.96410 | 3.03896 | 0.01976 | Root_Mass b_dist |
| Genons(Day_14,df=2)1 | 8.00000 | 8,723.71652 | 1,090.46457 | 1.96673 | 0.05586 | Root_Mass b_dist |
| Residuals8 | 126.00000 | 69,861.25916 | 554.45444 | NA | NA | Root_Mass b_dist |
| Geno8 | 4.00000 | 176,944.42509 | 44,236.10627 | 24.22780 | 0.00000 | Root_Mass b_sylv |
| Residuals9 | 133.00000 | 242,836.80767 | 1,825.84066 | NA | NA | Root_Mass b_sylv |
| Geno9 | 4.00000 | 2.81680 | 0.70420 | 36.70369 | 0.00000 | Shoot_Root_Ratio b_dist |
| ns(Day_14,df=2)6 | 2.00000 | 0.15610 | 0.07805 | 4.06809 | 0.01925 | Shoot_Root_Ratio b_dist |
| Harv7 | 4.00000 | 1.14490 | 0.28623 | 14.91840 | 0.00000 | Shoot_Root_Ratio b_dist |
| Residuals10 | 135.00000 | 2.59012 | 0.01919 | NA | NA | Shoot_Root_Ratio b_dist |
| Geno10 | 4.00000 | 2.15066 | 0.53767 | 58.14315 | 0.00000 | Shoot_Root_Ratio b_sylv |
| I(Day_14{}2)7 | 1.00000 | 0.04624 | 0.04624 | 5.00027 | 0.02713 | Shoot_Root_Ratio b_sylv |
| Harv8 | 4.00000 | 0.38527 | 0.09632 | 10.41571 | 0.00000 | Shoot_Root_Ratio b_sylv |
| GenoI(Day_14{}2)2 | 4.00000 | 0.13787 | 0.03447 | 3.72728 | 0.00671 | Shoot_Root_Ratio b_sylv |
| Residuals11 | 124.00000 | 1.14666 | 0.00925 | NA | NA | Shoot_Root_Ratio b_sylv |
| Geno11 | 4.00000 | 312,787.50625 | 78,196.87656 | 18.62845 | 0.00000 | biomass b_dist |
| ns(Day_14,df=2)7 | 2.00000 | 147,377.96199 | 73,688.98099 | 17.55455 | 0.00000 | biomass b_dist |
| Harv9 | 4.00000 | 145,877.82793 | 36,469.45698 | 8.68793 | 0.00000 | biomass b_dist |
| Residuals12 | 135.00000 | 566,691.30055 | 4,197.71334 | NA | NA | biomass b_dist |
| Geno12 | 4.00000 | 862,484.81413 | 215,621.20353 | 24.16680 | 0.00000 | biomass b_sylv |
| Harv10 | 4.00000 | 98,371.42927 | 24,592.85732 | 2.75636 | 0.03065 | biomass b_sylv |
| Residuals13 | 129.00000 | 1,150,964.71805 | 8,922.20712 | NA | NA | biomass b_sylv |
| I(Day_14{}2)8 | 1.00000 | 9,393.36268 | 9,393.36268 | 4.44262 | 0.03680 | c_content b_dist |
| Residuals14 | 143.00000 | 302,355.81423 | 2,114.37632 | NA | NA | c_content b_dist |
| Geno13 | 4.00000 | 16,887.94249 | 4,221.98562 | 4.20111 | 0.00314 | c_content b_sylv |
| ns(Day_14,df=2)8 | 2.00000 | 3,997.44433 | 1,998.72217 | 1.98884 | 0.14106 | c_content b_sylv |
| Residuals15 | 128.00000 | 128,635.92473 | 1,004.96816 | NA | NA | c_content b_sylv |
| Geno14 | 4.00000 | 14.72235 | 3.68059 | 24.76573 | 0.00000 | d13c b_dist |
| I(Day_14{}2)9 | 1.00000 | 4.81710 | 4.81710 | 32.41303 | 0.00000 | d13c b_dist |
| Harv11 | 4.00000 | 7.60700 | 1.90175 | 12.79639 | 0.00000 | d13c b_dist |
| Residuals16 | 135.00000 | 20.06317 | 0.14862 | NA | NA | d13c b_dist |
| Geno15 | 4.00000 | 12.87800 | 3.21950 | 14.09960 | 0.00000 | d13c b_sylv |
| ns(Day_14,df=2)9 | 2.00000 | 3.81605 | 1.90802 | 8.35607 | 0.00039 | d13c b_sylv |
| Harv12 | 4.00000 | 25.16097 | 6.29024 | 27.54772 | 0.00000 | d13c b_sylv |
| Residuals17 | 124.00000 | 28.31414 | 0.22834 | NA | NA | d13c b_sylv |
| Geno16 | 4.00000 | 332.26225 | 83.06556 | 8.93282 | 0.00000 | n_content b_dist |
| I(Day_14{}2)10 | 1.00000 | 15.55323 | 15.55323 | 1.67259 | 0.19812 | n_content b_dist |
| Harv13 | 4.00000 | 139.69914 | 34.92478 | 3.75579 | 0.00626 | n_content b_dist |
| Residuals18 | 135.00000 | 1,255.35355 | 9.29892 | NA | NA | n_content b_dist |
| Geno17 | 4.00000 | 456.11421 | 114.02855 | 25.06977 | 0.00000 | n_content b_sylv |
| ns(Day_14,df=2)10 | 2.00000 | 325.38436 | 162.69218 | 35.76872 | 0.00000 | n_content b_sylv |
| Genons(Day_14,df=2)2 | 8.00000 | 145.98218 | 18.24777 | 4.01187 | 0.00030 | n_content b_sylv |
| Residuals19 | 120.00000 | 545.81385 | 4.54845 | NA | NA | n_content b_sylv |
| Geno18 | 4.00000 | 14.25665 | 3.56416 | 5.34810 | 0.00050 | d15n b_dist |
| ns(Day_14,df=2)11 | 2.00000 | 14.26839 | 7.13419 | 10.70500 | 0.00005 | d15n b_dist |
| Harv14 | 4.00000 | 21.75070 | 5.43768 | 8.15934 | 0.00001 | d15n b_dist |
| Residuals20 | 134.00000 | 89.30239 | 0.66644 | NA | NA | d15n b_dist |
| I(Day_14{}2)11 | 1.00000 | 17.12283 | 17.12283 | 32.60495 | 0.00000 | d15n b_sylv |
| ns(Day_14,df=2)12 | 2.00000 | 2.16282 | 1.08141 | 2.05920 | 0.13179 | d15n b_sylv |
| Harv15 | 4.00000 | 13.73616 | 3.43404 | 6.53903 | 0.00008 | d15n b_sylv |
| Residuals21 | 127.00000 | 66.69536 | 0.52516 | NA | NA | d15n b_sylv |
| Geno19 | 4.00000 | 434.41850 | 108.60463 | 18.40460 | 0.00000 | c_n b_dist |
| I(Day_14{}2)12 | 1.00000 | 79.87162 | 79.87162 | 13.53538 | 0.00034 | c_n b_dist |
| Harv16 | 4.00000 | 75.29789 | 18.82447 | 3.19007 | 0.01537 | c_n b_dist |
| Residuals22 | 135.00000 | 796.62842 | 5.90095 | NA | NA | c_n b_dist |
| Geno20 | 4.00000 | 1,484.86836 | 371.21709 | 40.40652 | 0.00000 | c_n b_sylv |
| I(Day_14{}2)13 | 1.00000 | 488.17121 | 488.17121 | 53.13683 | 0.00000 | c_n b_sylv |
| GenoI(Day_14{}2)3 | 4.00000 | 90.41398 | 22.60349 | 2.46036 | 0.04877 | c_n b_sylv |
| Residuals23 | 125.00000 | 1,148.38252 | 9.18706 | NA | NA | c_n b_sylv |

Table S2:

*Selected model r-squared and AIC values*

| trait | sp | r2 | AIC |
| --- | --- | --- | --- |
| Relative_WC | b_dist | 0.644 | 809.716 |
| Relative_WC | b_sylv | 0.460 | 725.960 |
| SLA | b_dist | 0.698 | 851.463 |
| SLA | b_sylv | 0.304 | 824.383 |
| aboveground_greenarea | b_dist | 0.588 | 2,484.703 |
| aboveground_greenarea | b_sylv | 0.444 | 2,522.983 |
| Shoot_Mass | b_dist | 0.516 | 1,550.356 |
| Shoot_Mass | b_sylv | 0.478 | 1,503.130 |
| Root_Mass | b_dist | 0.548 | 1,357.246 |
| Root_Mass | b_sylv | 0.422 | 1,434.886 |
| Shoot_Root_Ratio | b_dist | 0.614 | -150.328 |
| Shoot_Root_Ratio | b_sylv | 0.703 | -239.448 |
| biomass | b_dist | 0.517 | 1,644.869 |
| biomass | b_sylv | 0.455 | 1,657.609 |
| c_content | b_dist | 0.030 | 1,525.673 |
| c_content | b_sylv | 0.140 | 1,325.141 |
| d13c | b_dist | 0.575 | 146.704 |
| d13c | b_sylv | 0.596 | 196.255 |
| n_content | b_dist | 0.280 | 746.466 |
| n_content | b_sylv | 0.630 | 603.709 |
| d15n | b_dist | 0.360 | 365.210 |
| d15n | b_sylv | 0.331 | 305.920 |
| c_n | b_dist | 0.425 | 680.522 |
| c_n | b_sylv | 0.642 | 694.126 |

Figure S1: Distributions of (a) *B. distachyon* and (b) *B. sylvaticum* reported on GBIF as of 2019.18.08. Examples of (c) *B. distachyon* Carly Slawson (CC BY 4.0, <https://www.inaturalist.org/photos/42532397>) and (d) *B. sylvaticum* Grzegorz Grzejszczak (CC BY-NC 4.0, <https://www.inaturalist.org/photos/36088991>) (GBIF.org (26 February 2020) GBIF Occurrence Download <https://doi.org/10.15468/dl.rau5v9>). Note that “B. distachyon” entries in GBIF may not distinguish among newly recognized annual species *B. distachyon s.s.*, *B. stacei*, and *B. hybridum* (*sensu* Catalan *et al*. 2012. Ann. Botany 109: 385-405).


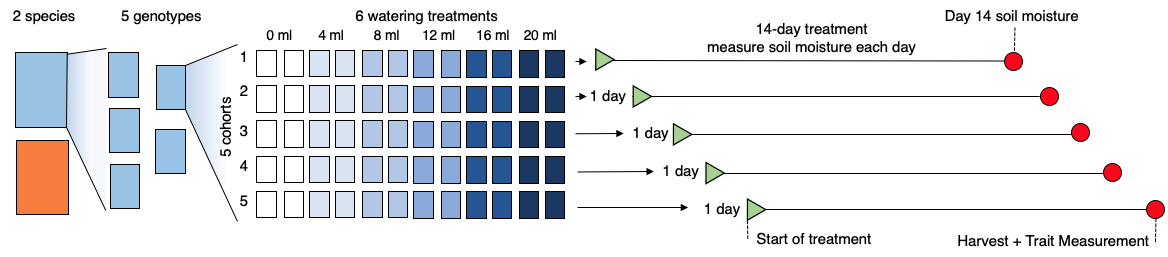


Fig S2: Experimental design. Shown is the schema for each of the 10 genotypes.


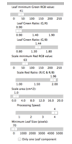


Fig S3: Settings used in Easy Leaf Area.

Fig S4: Variation in pot field capacity.

Figure S5: Plasticity through multivariate trait space. Principal component analysis of scaled phenotypic responses to soil moisture gradient among genotypes of both species. Upper panels show genotype means across soil moisture content. Percent values in axis titles indicate percent variance explained by that principal component. Lower panels show eigenvectors of each trait. (a) PC1 and PC2. (b) PC3 and PC4. *B. sylvaticum* is colored in orange and *B. distachyon* blue.
